# Applying Differential Learning During Rehabilitation After Anterior Cruciate Ligament Injury: A Basketball Single-Case Study

**DOI:** 10.3390/healthcare13243247

**Published:** 2025-12-11

**Authors:** Jorge Arede, Rui Zhou, Harjiv Singh, Wolfgang I. Schöllohrn

**Affiliations:** 1 School of Education, Polytechnic Institute of Viseu, 3504-510 Viseu, Portugal; jorge_arede@hotmail.com; 2 Department of Sports, Exercise and Health Sciences, University of Trás-os-Montes and Alto Douro, 5001-801 Vila Real, Portugal; 3 Research Center in Sports Sciences, Health Sciences and Human Development, CIDESD, 5001-801 Vila Real, Portugal; 4 Research Group on Physical Activity, Education, and Health (GIAFES), Universidad Católica de Ávila, C/Canteros, 05005 Ávila, Spain; 5 School of Sports Management and Communication, Capital University of Physical Education and Sports, Beijing 100191, China; 6 Human Performance and Sport Science Center, University of Michigan, Ann Arbor, MI 48109, USA; singh.harjiv@gmail.com; 7 Charlotte Hornets Basketball Club, Charlotte, NC 28202, USA; 8 Institute of Sport Science, Training and Movement Science, University of Mainz, 55128 Mainz, Germany; wolfgang.schoellhorn@uni-mainz.de

**Keywords:** motor learning, neuromuscular, return-to-play, movement variability, sensorimotor integration, anterior cruciate ligament injury, rehabilitation

## Abstract

**Background/Objectives**: Differential learning (DL) amplifies natural fluctuations in movement execution and, in its more extreme forms, facilitates repetition-free training with minimal external feedback. While increasingly recognized in the field of skill acquisition, its application in anterior cruciate ligament (ACL) rehabilitation remains underexplored. **Methods**: This study examined the application of DL in the rehabilitation of an 18-year-old trained basketball player following left-ACL reconstruction. The athlete completed a 42-week rehabilitation program in which DL principles were incorporated throughout the pre-operative, early, mid-, and late phases, culminating in return to sport. Training included differential mobility work, motor control, plyometric exercises, and sport-specific drills. Functional recovery was evaluated using single-leg hop tests, change-of-direction tasks, and sprint performance, while self-reported knee function was monitored via the International Knee Documentation Committee (IKDC) questionnaire. **Results**: Results indicated substantial improvements in both functional performance and psychological readiness. The IKDC score increased from 13.8% at baseline to 95.4% postoperatively, reaching the normal functional range. An ACL-RSI score of 85.2%, and inter-limb asymmetries were reduced to below 10%. Strength, agility, and sprint performance exceeded pre-injury levels. **Conclusions**: DL again shows potential as an effective approach to facilitating recovery and return to sport after ACL reconstruction, but larger controlled studies are needed for validation.

## 1. Introduction

Anterior cruciate ligament (ACL) injuries are among the most prevalent and debilitating conditions in sports [1,2]. Although ACL reconstruction (ACLR) techniques continue to advance and rehabilitation protocols have become increasingly standardized, only about 65% of athletes successfully return to their pre-injury level of competition, and nearly 20% experience a secondary ACL injury [3,4]. This phenomenon suggests that current rehabilitation systems have yet to sufficiently address the comprehensive recovery needs of athletes following ACL injury. The complexity of ACL rehabilitation lies in the need to simultaneously address biomechanical deficits, neuromuscular impairments, and psychological readiness—all of which influence recovery outcomes [5]. However, the latter remains insufficiently incorporated into traditional rehabilitation programs [6]. Conventional rehabilitation approaches emphasize uniformity and repetition, focusing on achieving quantifiable milestones such as range of motion (ROM), maximal muscle strength, and single-leg hop symmetry [7,8]. While these indicators are undoubtedly important, emerging evidence indicates that such standardized methods may not adequately prepare athletes for the unpredictability and variability of real sporting environments [9]. Therefore, given the coexistence of physiological and psychological demands, current ACL rehabilitation strategies still require further optimization.

Differential Learning (DL) is an innovative paradigm that aligns closely with the inherent diversity and adaptability required in sports. DL emphasizes the integration of movement variability and cognitive engagement during training, breaking away from the repetitive practice and corrective strategies characteristic of traditional methods [10]. By amplifying fluctuations in movement execution, DL enhances motor learning efficiency, strengthens neuromuscular control, and improves athletes’ perceptual responsiveness and resilience in dynamic environments—factors that may contribute to reduced injury risk [11,12]. Based on these features, DL can be considered a potential supplement to existing ACL rehabilitation frameworks.

As noted earlier, common post-ACLR issues such as abnormal knee joint kinematics and quadriceps weakness increase the risk of re-injury [13]. Furthermore, current evidence indicates that if perceptual and neuromuscular control capacities are not adequately trained during rehabilitation, athletes may display deficits in perception–action coupling and neuromuscular coordination when returning to complex sporting scenarios—even when strength and ROM have been restored [14]. Traditional rehabilitation programs mainly focus on restoring biomechanical and basic physical capacities in stable, controlled, and predictable settings, which makes it difficult to simulate the highly dynamic, rapidly changing, and unpredictable environments athletes face upon returning to their sport [15]. Although traditional programs introduce limited variability or uncertainty training in later stages, they still rely primarily on pre-planned exercises and emphasize repetitive practice and error correction, largely independent of individual movement solutions [16]. These characteristics are considered potential contributors to secondary injury. In contrast, DL represents a fundamentally different training paradigm, characterized by the systematic introduction of variability throughout the entire process [10]. This approach is believed to enhance movement coordination on top of restoring fundamental strength and mobility, enabling individuals to better cope with changes in sports environments [17].

The advantages of DL extend beyond the physiological aspects and demonstrate potential benefits in psychological recovery. Athletes recovering from ACL often experience fear of re-injury, reduced confidence, and anxiety about returning to sport [18]. The cognitive demands of DL exercises with its implicit characteristics may help redirect attention from the injury to task execution, fostering psychological resilience [19]. Preliminary evidence suggests that DL can enhance an athlete’s perception of control and competence, contributing to improved psychological readiness [10]. In another study, the DL group demonstrated more structured mental representations following the intervention. This enhanced organization of mental representation is believed to support greater psychological adaptability and strategy transfer capabilities [20]. These findings suggest that DL could be a valuable strategy for optimizing the ACL rehabilitation process and enhancing the quality of athletes’ psychological recovery.

Despite its theoretical advantages, the application of DL in ACLR is relatively unexplored. Existing research has primarily focused on its use in healthy populations or skill acquisition contexts, with limited data on its integration into clinical settings. Although a recent randomized controlled trial indicated that DL-based interventions could reduce biomechanical risk factors for ACL injuries in athletes, further research is still required to confirm its effectiveness in ACLR [14].

This case study explores the feasibility and effectiveness of integrating DL principles into a structured rehabilitation program for an amateur basketball player recovering from ACLR. The participant, an 18-year-old male, sustained a complete ACL tear during a basketball game and underwent surgical reconstruction using a patellar tendon allograft. The 42-week rehabilitation program incorporated DL principles throughout the pre-operative, early rehabilitation, mid-rehabilitation, late rehabilitation, and return-to-sport phases, with key objectives centered on restoring knee joint stability, enhancing functional performance, and strengthening psychological readiness for competition.

The study’s primary outcomes were assessed through functional and biomechanical measures, including bilateral and unilateral jump tests, change of direction (COD) quality, and sprint performance. Self-reported knee function was evaluated using the International Knee Documentation Committee (IKDC) questionnaire, while return-to-play readiness was measured with the ACL Return to Sport after Injury (ACL-RSI) score scale. By analyzing these key performance indicators, this study seeks to provide valuable insights into the potential benefits of DL in addressing the multifaceted challenges of ACL rehabilitation, offering a foundation for future research and clinical applications in sports injury recovery.

## 2. Detailed Case Description

### 2.1. Participant

A trained male basketball player (age: 18 years; height: 1.70 m; weight: 75 kg; BMI = 26 kg/m^2^) participated in this study. The subject was early specialized in basketball, having 12 years of basketball experience and 5 years of structured resistance training. At the moment of the injury, the subject participated in 2 strength and conditioning sessions, 4 basketball sessions, and 1 competitive game per week. Their training process included regular monitoring of physical abilities. Test results before the ACL injury are shown in Table 1. Written informed consent was obtained from the participant, and the study adhered to institutional ethical guidelines.

### 2.2. Rehabilitation Protocol

The player underwent a 42-week rehabilitation program, incorporating DL principles across distinct rehabilitation phases. The team of independent physiotherapists conducted the rehabilitation. In parallel with the traditional rehabilitation protocol, DL was integrated throughout all phases of rehabilitation. This process requires athlete to perform various movement variations, which are selected according to the training principles of DL (Table 2). For example, in terms of geometric variation, “looking to the right” means that the athlete maintains the head turned to the right during the exercise (e.g., keeping the head oriented to the right while performing a squat), thereby altering visual input and cervical posture and requiring the body to adjust trunk and lower-limb coordination to maintain balance and directional control. For velocity-based variation, “right hip faster than left hip” indicates that the athlete deliberately increases the movement speed of the right hip during the exercise (e.g., intentionally accelerating right-hip force production or movement speed during a forward lunge), creating a temporal asymmetry between sides and inducing a coordination perturbation. These variations are used to promote self-organization under diverse conditions, consistent with the core principles of DL [21].

The rehabilitation program was divided into six phases: (i) pre-operative, (ii) early rehabilitation, (iii) mid-rehabilitation, (iv) late rehabilitation, (v) return to training, (vi) return to play (Table 3). Progression between phases was contingent on meeting predefined criteria (Table 3), based on previous studies [22].

#### 2.2.1. Pre-Operative Phase

In the sixth month of the competitive season, during a match from the 4th division of the National Senior Championship in the evening, the athlete sustained an ACL rupture in the non-dominant limb (left leg) due to a contact-related mechanism while executing a lay-up. Upon initial ground contact, the player experienced a forceful collision with a defender, which led to an uncontrolled landing on the second contact, with the knee fully extended. Subsequent magnetic resonance imaging (MRI) of the knee one day after the injury revealed an interruption of most fibers within the anterior cruciate ligament, indicative of a high-grade injury. The external meniscus exhibited signs of a posterior horn rupture. While the internal collateral ligament was slightly thickened and presented with surrounding effusion, the apparent integrity of its fibers suggested a low-grade injury, likely a sprain. The internal meniscus demonstrated preserved normal morphology and signal intensity. Both the posterior cruciate and external collateral ligaments were intact. The quadriceps tendon and patellar ligament showed no abnormalities. A peri centimeter contusion was noted on the posterior aspect of the external tibial plateau. Moderate joint effusion and effusion within the suprapatellar bursa were also observed. These findings are suggestive of a significant ACL injury, possibly a complete tear, along with damage to the external meniscus. The internal collateral ligament appears to be involved, though likely to a lesser degree.

Following the MRI scan, which confirmed the diagnosis of the injury, the preoperative phase commenced with the primary objective of reducing inflammation, swelling, and pain. Once the diagnosis was established, surgical intervention was scheduled.

**Figure 1:**
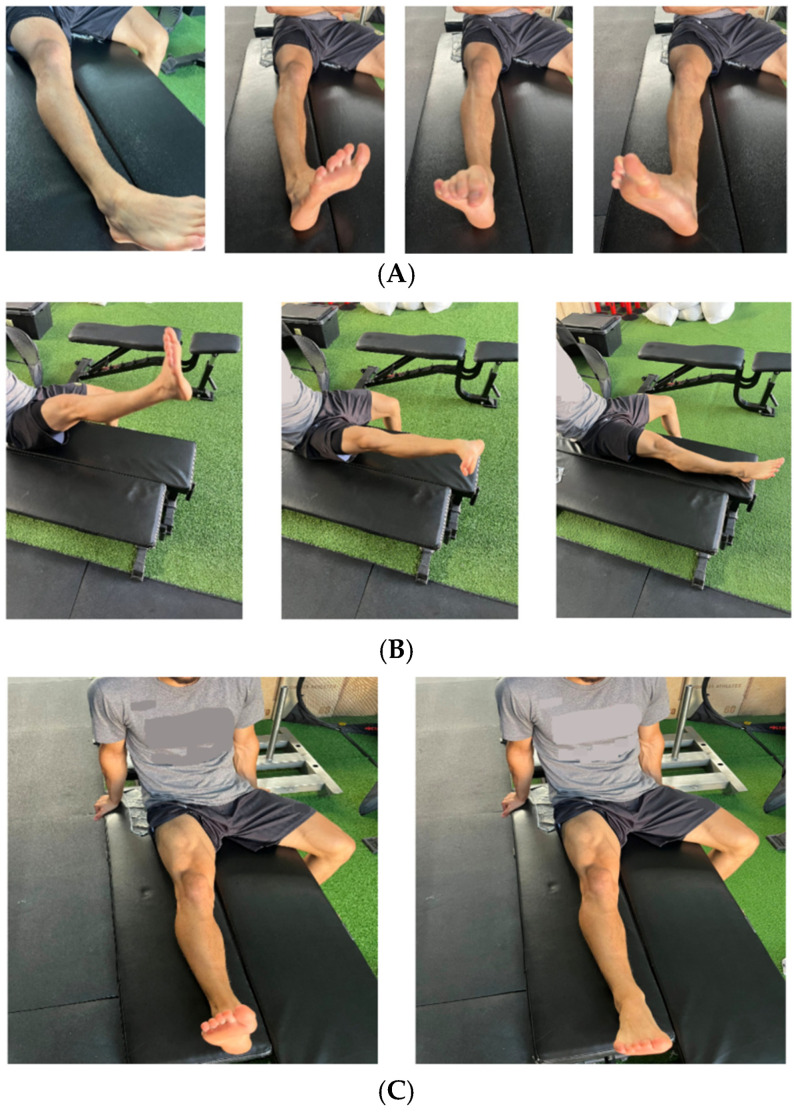
(**A**) Example of motor control exercise implemented during the early postoperative rehabilitation phase: ankle pumps. (**B**) Example of motor control exercise implemented during the early postoperative rehabilitation phase: single leg raises. (**C**) Example of motor control exercise implemented during the early postoperative rehabilitation phase: quadriceps set. (Note: Photographs were taken retrospectively for illustrative purposes and do not represent the clinical setting or real-time execution of the rehabilitation exercises).

In the initial phase of rehabilitation, mobility exercises (e.g., passive knee extension to 0° and passive knee flexion to tolerance) and motor control exercises (e.g., ankle pumps, quadriceps setting, and straight leg raises) were implemented. Additionally, differential walking, based on modifications in movement geometry, was introduced. These exercises were performed with varying repetitions, exploring different degrees of freedom across multiple joints and movement planes (e.g., ankle dorsiflexion and plantarflexion, inversion and eversion, hip internal and external rotation, as well as various degrees of knee flexion) (Figure 1).

**Figure 2:**
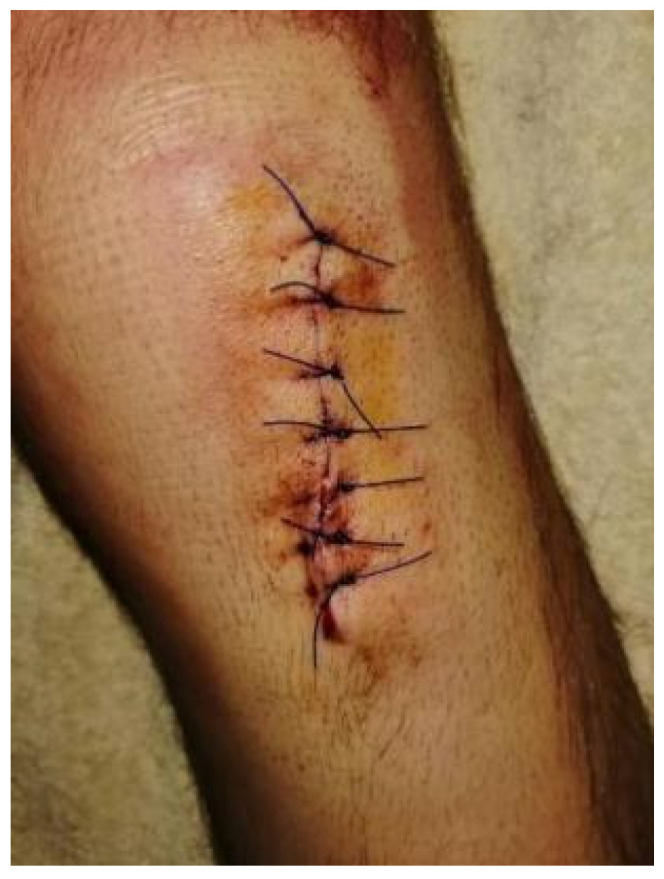
Surgical reconstruction using a patellar tendon allograft 25 days post-injury.

As the rehabilitation progressed, more advanced functional patterning exercises were incorporated, including mini squats, lunges, step-ups, hip external and internal rotation, retro stepping drills, and balance training drills. These exercises followed the same principle of modifying movement geometry, ensuring variability in execution, forcing all muscles around the knee and neighboring joints to interact under various gravitational and inertial forces. During walking exercises, intentional alterations in head, arm, trunk, and hand positioning were introduced to enhance movement adaptability. Upper-body strength training was maintained during this phase to support general conditioning and enhance fatigue resistance [23]. Surgical reconstruction was performed 25 days post-injury, utilizing a patellar tendon allograft (Figure 2), after all necessary surgical criteria had been met, as confirmed by the surgeon.

#### 2.2.2. Early Rehabilitation Phase

Following the successful completion of surgery, the early rehabilitation phase commenced. The initial 17 days of this phase consisted of the immediate postoperative period, primarily aimed at restoring full passive knee extension. During this sub-phase, a range of mobility exercises (e.g., active and passive knee flexion up to 90° in sitting, laying prone or sidewards and standing position, hamstring stretches and standing hamstring curls) and motor control exercises (e.g., ankle pumps, quadriceps setting, straight leg raises, knee extension from 90° to 40°, multi-angle isometrics at 90° and 60° for knee extension) were implemented. Additionally, functional patterning exercises (e.g., mini squats and weight shifts) were introduced, following the principles of DL by incorporating movement geometry variations, as previously described in the preoperative phase.

Upon meeting the criteria (Table 3) the early rehabilitation phase began, lasting 12 days. This phase introduced further mobility exercises (e.g., standing hamstring curls, PROM from 0° to 100°, well-leg exercises, and PROM from 0° to 105°), motor control exercises (e.g., quadriceps setting, straight leg raises, knee extension from 90° to 40°, and an eccentric quadriceps program from 40° to 100°), and functional patterning exercises (e.g., half squats from 0° to 40°, weight shifts, front lunges, side lunges, squats on a tilt board, front step-downs, and lateral step-overs, squats with different speed during up and downward movement), all aligned with the DL approach. The upper-body strength training routines were maintained throughout this phase. Additionally, fatigue resistance exercises were also preserved.

By the fourth week, bicycle exercise was introduced to facilitate the restoration and maintenance of full passive knee extension (≥0° to 5–7° of hyperextension). This phase ensured the achievement of the necessary criteria to progress to the subsequent rehabilitation stage (Table 3).

#### 2.2.3. Mid-Rehabilitation Phase

This phase lasted for 44 days and was primarily aimed at achieving a full knee ROM from 0° to 125°. During this period, there was an increase in both the number of exercises performed and the time dedicated to functional patterning exercises.

Mobility exercises primarily consisted of hamstring curls, while motor control exercises included knee extension exercises performed from 90° to 40° of flexion, hip abduction and adduction, and hip flexion and extension. The functional patterning exercises incorporated lateral step-overs, side lunges, lateral step-ups, front step-downs, wall squats, vertical squats, standing toe calf raises, seated toe calf raises, balance exercises, and ball throws. All exercises were structured according to the principles of DL, emphasizing variations in movement geometry.

During the first three weeks of this phase, bicycle training was repurposed for aerobic conditioning, with increased duration and intensity to improve cardiovascular endurance. Over the following three weeks, differential walking exercises were progressively implemented, with a weekly increase in both walking speed and the complexity of movement tasks. These exercises incorporated discrete and sequential movement patterns across different planes of motion. As the knee range of motion improved, additional exercises were incorporated into the upper-body strength training routines, including the bent-over row and pull-up. Additionally, fatigue resistance exercises designed to enhance repeated power output, particularly using the bench press, were also maintained. Progression to the next rehabilitation stage was determined based on the criteria described (Table 3).

#### 2.2.4. Late Rehabilitation Phase

**Figure 3:**
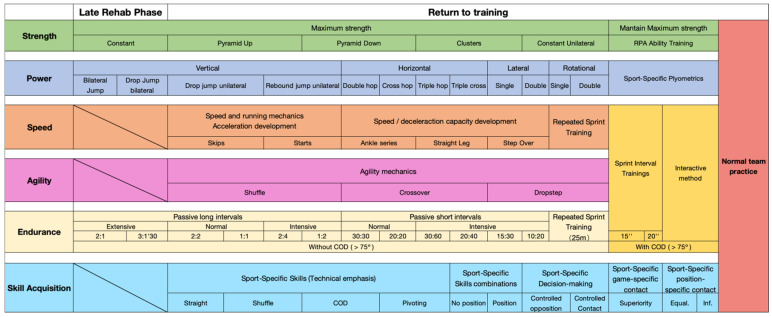
Late-phase maximum physical qualities training planning.

This phase lasted 66 days and was primarily aimed at normalizing lower extremity strength (Figure 3). During this period, the focus was on motor control exercises, including knee extension from 90° to 40°, hip abduction and adduction, and hip flexion and extension, as well as functional patterning exercises, such as lateral step-overs, side lunges, lateral step-ups, front step-downs, wall squats, vertical squats, standing toe calf raises, seated toe calf raises, balance exercises, and ball throws.

Differential walking continued to be emphasized during the first three weeks of this phase, incorporating variations in movement speed. By the fourth week, continuous running (15 min fartlek training) was introduced, also applying DL principles through variations in movement geometry.

At this stage, the athlete was already completing the full upper-body training routine, which included both maximum strength exercises and repeated power training [24].

During the final six weeks, a structured maximum strength development program was implemented using a constant-load method, focusing on bilateral lower-body exercises, particularly the squat and deadlift (Figure 3). These two exercises were extensively performed by the athlete before the injury, given their prior experience with resistance training.

Additionally, a plyometric training program was introduced, emphasizing bilateral exercises performed in the sagittal plane with a vertical movement pattern. Furthermore, interval training with long passive recovery periods was incorporated [25]. It is important to note that these training programs were performed at least twice per week with two days in between, ensuring that the training schedule and exercise order were structured to prevent interference effects. All exercises were conducted based on DL principles on the first level, incorporating movement geometry variations.

For the interval training program, maximal aerobic speed (MAS) was estimated using the final stage velocity of the Yo-Yo Intermittent Recovery Test Level 1 (Yo-Yo IR1), calculated as: VYo-Yo = V + 0.5 × (n/8), where V is the velocity of the penultimate stage and n the number of runs completed in the final stage [26]. Although the Yo-Yo IR1 is not a valid substitute for laboratory-based MAS tests due to limited agreement, it was used in this case for its practicality, the athlete’s test familiarity, and the availability of pre-injury data [27]. After accomplishing the next stage criteria, the athlete progressed to the subsequent phase (Table 3).

#### 2.2.5. Return to Training Phase

This phase lasted 109 days and consisted of three distinct sub-phases: Return to Sport-Specific Skills (51 days), Return to Reduced Basketball Practice (25 days), and Return to Full Basketball Practice (33 days).

(1) Return to Sport-Specific Skills sub-phase

During the first sub-phase, the maximal strength training initiated in the previous phase was continued, now incorporating additional training methods (Figure 3). In the final three weeks, unilateral maximal strength exercises, such as the Split Squat and Lateral Squat, were introduced. Regarding plyometric training that corresponds to the DL principle of variations in velocity and acceleration, new exercises were implemented that involved movements across different planes, along with a progressive increase in axial loading.

All exercises were performed following the principles of DL, emphasizing variations in movement geometry, velocity, acceleration, and rhythm [28]. This sub-phase also marked the introduction of a speed training program. Initially, the focus was on running technique, incorporating variations in movement geometry and velocity. In the latter stage, repeated sprint training was integrated twice per week, also incorporating movement geometry and velocity variations.

For agility development, two weeks were dedicated to each of the primary techniques, consistently applying DL principles (movement geometry). In terms of endurance training, high-intensity interval training (HIIT) continued to be utilized, incorporating different interval types and work-to-rest ratio combinations. In the final phase, endurance training coincided with speed training sessions. Notably, changes in direction exceeding 75° were deliberately avoided during this phase.

A strong emphasis was placed on skill acquisition based on DL principles. Initially, discrete skills were trained before gradually being integrated into more complex sequences. Skills evolved in terms of position specificity and progressively introduced controlled physical contact. Progression to the next sub-phase occurred upon meeting the defined criteria (Table 3).

(2) Return to Basketball Practice sub-phase

During this sub-phase, the primary goal of maximal strength training was to maintain strength levels using the repeated power method, a method that maximizes propulsive power output in which the athlete was already well-trained [24,29]. This method included movement geometry variations as documented in the literature. Training was conducted three times per week before the team practice sessions, following a standardized warm-up.

A basketball-specific plyometric training program was implemented, incorporating work with the ball and emphasizing different movement possibilities associated with the “gather step” technique. Various movement geometry adaptations were included to further enhance neuromuscular stimulus.

For speed, agility, and endurance training, Sprint Interval Training (SIT) was employed during the first two weeks, followed by the Interactive Method in the final two weeks (Figure 3). This work was performed during sessions in which the athlete was not participating in team training or skill acquisition drills. Throughout this sub-phase, more aggressive directional changes (>75°) were introduced.

Regarding skill acquisition, the athlete initially participated in superiority-based drills (e.g., 2v1), with free contact but without position specificity (Figure 3). Gradually, position-specific drills were introduced, followed by even-numbered (e.g., 3v3) and disadvantage-based situations (e.g., 2v3) [30]. Advancement to the next sub-phase was contingent upon meeting the defined criteria (Table 2). Upon satisfying these benchmarks, the athlete resumed full, unrestricted team training. At this stage, the athlete demonstrated maximal strength levels in both squat and bench press that exceeded pre-injury competition levels. In the final sub-phase, the athlete was fully reintegrated into regular team training without restrictions.

(3) Return-to-play phase

After meeting the criteria to train freely with the team for 33 days (i.e., Return to Basketball Practice sub-phase), the athlete returned to competition in a game at the same pre-injury level on 5 February 2023. However, the match was of low difficulty and highly unbalanced in favor of the athletes’ team, with a 31-point difference. In concrete terms, the athlete played 32 min, recorded 4 assists, and grabbed 3 rebounds in a low-difficulty game.

### 2.3. Monitoring

Before the injury, the athlete regularly underwent a series of tests to gain a better understanding of their physical performance, as described elsewhere [24]. During the rehabilitation process, additional physical assessments were conducted to more accurately evaluate the criteria for progression to the next stage. Both the criteria and the tests are well-established references in scientific literature dedicated to ACL injury rehabilitation protocols.

From the day of injury, self-reported knee function was assessed each morning using the International IKDC questionnaire via an online form. IKDC scores ranged from 0 to 100, with higher scores indicating better function. From the return-to-training phase onwards, a more comprehensive monitoring process was implemented to ensure appropriate adaptation.

Training load (TL) was quantified using session ratings of perceived exertion (sRPE) based on the Borg CR10 scale, recorded via an electronic form. Participants rated their exertion 30 min post-session, with weekly TL calculated as the sum of all session TLs. The Acute: Chronic Workload Ratio (ACWR) was determined using both rolling average and Exponentially Weighted Moving Average (EWMA) methods.

Daily monitoring included sleep tracking via a smart band, with sleep efficiency calculated as the ratio of total sleep time to time in bed. If efficiency fell below 85%, participants were advised to nap (20–90 min) between 1:00 and 4:00 PM [31].

Each morning, heart rate variability (HRV) and resting heart rate (RHR) were measured using HRV4Training (A.S.M.A. B.V., version 8.5.8, Amsterdam, The Netherlands), a validated mobile app requiring no external sensors. HRV4Training has been validated against electrocardiograms (ECG), demonstrating high accuracy via smartphone-based photoplethysmography. Participants submitted a screenshot of their HRV and RHR data, followed by completing the Total Quality Recovery (TQR) and IKDC questionnaires [32].

HRV was measured in a supine position using HRV4Training [33]. The weekly mean of the log-transformed square root of the mean sum of squared differences between consecutive R-R intervals (LnRMSSD) was used for analysis. Fatigue was indicated by decreased weekly HRV and increased RHR, while adaptation corresponded to increased HRV and decreased RHR.

The TQR scale (6–20) was used to assess recovery before the first daily training session. Based on TQR and HRV results, daily training adjustments were made in consultation with the athlete, modifying session volume and intensity as needed.

Importantly, the participant did not experience any major complications that could have affected the rehabilitation process, and overall adherence was satisfactory.

### 2.4. Statistical Analysis

An initial linear regression model was used to explore IKDC score trajectories but revealed violations of key assumptions, including autocorrelation (Durbin–Watson test), heteroscedasticity (Breusch–Pagan test), and non-normal residuals (Shapiro–Wilk and Lilliefors tests). To address temporal dependencies, autoregressive integrated moving average (ARIMA) modeling was applied following Box–Jenkins methodology. Model selection was based on AIC and BIC, with parameter significance tested using t-statistics. Residual diagnostics (Ljung–Box test, mean error, residual ACF, and variance) and forecast accuracy metrics (RMSE, MAE, MAPE) were used to assess model adequacy. Structural changes in the data were evaluated using Bai–Perron breakpoint analysis, supported by CUSUM and Chow tests. To model nonlinear recovery patterns, segmented regression with automatic breakpoint detection was employed, allowing for estimation of slope changes across distinct phases. Competing models, including linear, polynomial, ARIMA, and segmented approaches, were compared using AIC, RMSE, and explained variance to identify the most parsimonious and clinically interpretable model. Additionally, self-reported IKDC outcomes were analyzed concerning published normative reference data from healthy, age-matched populations. Statistical analysis was performed using R (2023.06.0), with significance set at *p* < 0.05.

### 2.5. Results

A comprehensive longitudinal analysis of IKDC score trajectories was conducted using a multi-method analytical strategy designed to accommodate temporal dependencies and potential nonlinearities. Initial linear regression highlighted significant assumption violations, including pronounced autocorrelation (Durbin–Watson statistic = 0.204, *p* < 2.2 × 10^−16^), heteroscedasticity (Breusch–Pagan χ^2^ = 72.35, *p* < 2.2 × 10^−16^), and non-normally distributed residuals (Shapiro–Wilk W = 0.944, *p* = 5.47 × 10^−8^; Lilliefors D = 0.093, *p* = 3.32 × 10^−5^). These findings necessitated a shift towards more robust time series methodologies. To address the temporal structure of the data, an autoregressive integrated moving average (ARIMA) model was applied. Model selection criteria (AIC and BIC) identified ARIMA(0,1,1) with drift as the optimal specification (AIC = 1221.43, BIC = 1231.87). Model estimation yielded a statistically significant moving average term (θ = −0.131 ± 0.065, t = −2.03, *p* = 0.043), alongside a positive drift component (β = 0.339 ± 0.171, t = 1.98, *p* = 0.048), indicating an underlying gradual improvement trend. Residual diagnostics supported the model’s adequacy, with white noise characteristics (Ljung–Box Q = 1.62, *p* = 0.996, df = 9), negligible bias (mean error = 0.0012), absence of residual autocorrelation (ACF_1_ = 0.0007), and homoscedasticity (σ^2^ = 9.344). Forecasting accuracy was also high, as reflected in RMSE = 3.038, MAE = 1.134, and MAPE = 3.64%.

**Figure 4:**
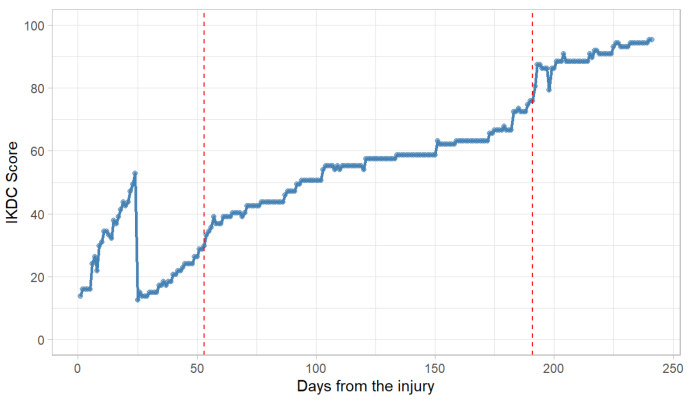
Temporal progression of IKDC scores following injury, analyzed using the Bai–Perron multiple breakpoint procedure. Note: Red dashed lines denote the estimated structural breakpoints.

To further examine temporal heterogeneity in recovery dynamics, structural break analysis was conducted using the Bai–Perron procedure. The algorithm identified up to three optimal breakpoints—at days 50, 114, and 191—based on minimized residual sum of squares (RSS = 6182 for three segments), with the two-breakpoint solution (days 53 and 191) yielding a substantial 41.5% reduction in residual variance relative to the initial linear model (Figure 4).

**Figure 5:**
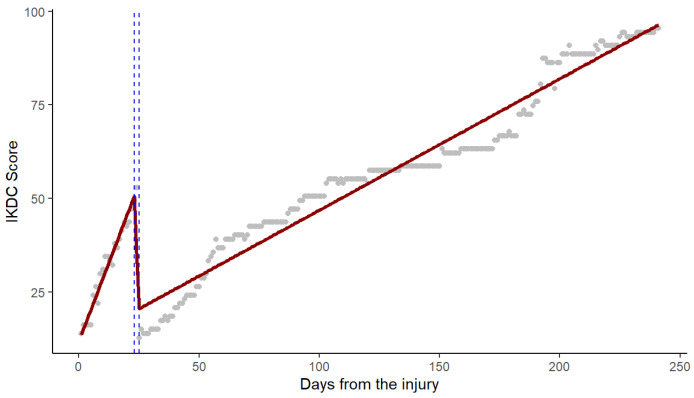
Piecewise linear regression model fitted to IKDC scores over time since injury. Note: Grey points represent the observed IKDC scores, red segmented lines denote the fitted piecewise linear regression model, and blue dashed vertical lines indicate the estimated breakpoints identified by the automated detection procedure.

This segmentation was supported by confirmatory CUSUM tests and a marginally non-significant Chow test (F = 2.84, *p* = 0.060), indicating structural instability in the trajectory. Subsequent piecewise regression modeling resolved prior violations of linear assumptions and provided a clinically interpretable framework. Automated breakpoint detection delineated three distinct recovery phases: an initial rapid improvement from days 0 to 23 (β = 1.682 ± 0.157), a brief transitional decline between days 23 and 25 marked by a sharp slope reversal (Δβ = −16.705 ± 3.299), and a post-transition phase of stabilized progression (Δβ = +15.375 ± 3.295) (Figure 5).

This segmented model demonstrated an excellent fit (R^2^ = 0.960, adjusted R^2^ = 0.959), substantial improvement in residual standard error (4.66; a 31.5% reduction versus linear), corrected autocorrelation (Durbin–Watson = 1.82, *p* = 0.12), and normally distributed residuals (Anderson–Darling A = 0.87, *p* = 0.08). In comparative performance analyses, the segmented regression approach outperformed both ARIMA and traditional linear models across multiple metrics: 12.4% lower AIC (1221.4 vs. 1393.6), 27.4% lower RMSE (4.66 vs. 6.81), and 4.3% higher explained variance compared to polynomial regression (96.0% vs. 91.7%). Collectively, these findings identify days 23–25 as a critical physiological transition window and substantiate the temporal dependency in recovery processes. While ARIMA modeling effectively captured autocorrelated structure, segmented regression provided superior interpretability and fit, successfully addressing initial modeling limitations.

At baseline, the player reported an IKDC score of 13.8% and a score of 52.9% one day before surgery. In the pre-operative phase, the subject reported values for IKDC above pre-operative values that occurred in another study [34]. One, two and three months after the injury, the IKDC value was below that reported after ACL injury observed in other studies [35,36]. The player achieved an acceptable symptom state threshold at day 190 (~6 months) after injury, and 6 months after surgery, reported knee function within normal ranges [7]. When the player was clinically assessed as ready to play competitively reported an ACL-RSI Score of 85.2% and IKDC of 95.4%, respecting criteria associated with a successful return to sport [37]. Moreover, the player revealed a Knee Outcome Survey Activities of Daily Living Scale of 95.7%, interlimb asymmetries below 10% in single-hop and triple-hop [38], but also better maximum strength, agility, and sprinting performance compared to the pre-injury period.

## 3. Discussion

The findings from this case study highlight the feasibility and potential benefits of integrating DL principles into the rehabilitation process following ACLR. By introducing movement variability into rehabilitation exercises, DL addresses critical deficits in neuromuscular control, proprioception, and psychological readiness, which are often persistent challenges after ACLR [10,13].

From a biomechanical perspective, insufficient lower-limb flexion—particularly limited hip flexion and ankle dorsiflexion—as well as increased knee valgus have been widely recognized as key contributors to ACL injury risk [39,40]. In this study, the athlete demonstrated inter-limb asymmetry below 10% during single-leg hop and landing tasks, along with notable improvements in knee control. These findings are consistent with existing evidence suggesting that correcting biomechanical asymmetries is critical for reducing the risk of secondary injuries [13].

Compared with traditional training approaches that emphasize fixed movement patterns, the key mechanism of DL lies in inducing movement variability through the introduction of stochastic perturbations (noise) [41]. Noise itself is not the training target but rather a means to encourage continuous adjustments in joint angles, muscle recruitment, and movement strategies throughout practice. When athletes actively explore movement solutions under multi-planar, multi-speed, and perturbed conditions, they naturally tend to increase lower-limb flexion, thereby reducing ground reaction forces (GRF) upon landing [42]. This creates a biomechanical foundation for mitigating high-risk compensatory patterns such as excessive knee valgus.

Movement variability is inherently an adaptive response influenced by factors such as emotional state [43], fatigue [44], and temporal dynamics [45]. Ideally, such variability should adjust to both internal and external perturbations, helping athletes avoid rigid and hazardous movement patterns during complex sport-specific tasks [46]. By leveraging this variability, DL enhances athletes’ tolerance to perturbations and facilitates the development of more robust movement control strategies through continuous exploration [10]. This mechanism aligns with prior research demonstrating the positive effects of DL on ACL injury prevention [14], further supporting DL as a promising and functionally advantageous rehabilitation approach.

Restoring neuromuscular control is one of the most critical challenges following ACLR [16]. In the early stages of injury and post-surgical recovery, the body instinctively redistributes muscular activity—both within and between muscles—to minimize pain and preserve short-term musculoskeletal integrity, often accompanied by neuromuscular inhibition [47,48]. While this inhibition serves as a protective mechanism, it may also lead to muscle atrophy, weakness, restricted joint range of motion, reduced movement variability, and an increased risk of re-injury, ultimately impairing athletic performance [49].

Traditional rehabilitation protocols primarily emphasize repetitive and symmetrical movements; however, such approaches may not adequately prepare athletes for the dynamic and unpredictable demands of sports [7]. In contrast, DL, which is grounded in principles of motor learning, system dynamics, and neurophysiology, places a stronger emphasis on movement variability, thereby fostering the adaptability and resilience of the neuromuscular system [14]. DL-based rehabilitation strategies introduce stochastic perturbations into training to facilitate implicit learning, encouraging the body to discover optimal movement solutions through self-organization [50]. In its most extreme form, DL-based rehabilitation eliminates repetitive elements and withholds explicit instructions about potential solutions, ensuring a truly self-organized learning process [28]. During this process, noise and perturbations are transmitted through mechanoreceptors, along with the vestibular and visual systems, providing broader input signals to neural networks across different brain regions. This, in turn, facilitates the formation of new and more efficient activation patterns and movement strategies [51].

Moreover, the emphasis on variability in DL may also enhance proprioceptive feedback, a critical function that is often impaired following ACLR [52]. Postoperative alterations in joint kinematics and diminished proprioceptive input significantly increase the risk of secondary injuries [7]. Further research has indicated that this risk is particularly elevated during the first two years following ACLR. In a prospective study involving 1432 athletes, the average time to ipsilateral ACL re-injury was 21.4 months postoperatively, while contralateral injuries occurred at an average of 24.6 months after surgery [53]. While patients may compensate by relying more heavily on visual feedback and consciously engaging cortical regions to optimize motor control, such compensatory strategies are primarily effective for simple movement tasks [54]. However, in sport-specific scenarios, such as COD tasks or reactive opponent-based training, these compensations may become ineffective, overloading the central nervous system and reducing movement efficiency, thereby increasing injury risk [55].

Therefore, exposing athletes to diverse movement patterns and training environments is essential for facilitating the reintegration of proprioceptive pathways and, ultimately, improving knee joint stability in a meaningful way. This reflects a core strategy of DL, namely enhancing movement variability throughout training [11]. Notably, ACL injuries most frequently occur during highly complex and reactive movement tasks rather than during pre-planned, controlled actions [56]. Compared with traditional training, many tasks performed within DL occur outside the athlete’s visual field, requiring reliance on proprioceptive and kinesthetic information to complete the movement. This implicitly reinforces the integration of perceptual and sensory feedback systems [57].

Traditional rehabilitation methods often fail to replicate the high-speed, multidirectional movements characteristic of sports such as basketball, potentially leaving athletes vulnerable to injury upon returning to competition [16]. Closed-skill practice is limited in its ability to adequately train the coupling between sensory cues and motor responses—an ability that is fundamentally shaped by environmental constraints and sport-specific demands [58]. Several ACL injury prevention and rehabilitation programs rely on preset instructional frameworks, often prescribing standardized movement execution patterns and landing postures [59].

In contrast, DL requires patients to continuously adapt their movement patterns throughout training, thereby developing individualized and adaptable movement solutions [60]. These movement variations are not indicative of poor performance or unstable skill execution; rather, they represent important adaptive characteristics that enhance movement proficiency [61]. From a complex systems perspective, DL functions as a form of functional adaptation in which variable instructions increase the likelihood of effectively conveying goal-relevant information, ultimately expanding the athlete’s repertoire of coordinated movement patterns and improving their ability to manage dynamic conditions [51]. This approach has also been applied successfully in motor skill learning [21].

As an essential component of the return to sport (RTS) continuum, individuals recovering from ACL injury should be progressively exposed to the physical, environmental, and psychological stressors characteristic of competitive sports [58]. Automatized landing techniques—performed without excessive conscious focus on achieving a “correct” landing posture—may be more advantageous for ACL recovery and injury prevention than repeatedly practicing rigid, predetermined movement patterns [1].

Athletes typically require 6 to 12 months to return to sport following ACLR, and an earlier return is associated with a higher risk of secondary injury [62,63]. Physical and biomechanical factors alone do not fully explain why athletes fail to RTS on time following ACLR [8]. Fear of re-injury and pain-related movement avoidance are among the primary psychological barriers to RTS, with studies indicating that up to 40% of athletes do not resume competitive sports due to psychological factors [6]. This psychological state has been linked to quadriceps weakness and lower self-reported functional levels, highlighting the need for a greater emphasis on the psychological impact on functional recovery after ACLR [64].

By simultaneously engaging cognitive and motor systems, DL may help mitigate these psychological barriers. The athlete in this study exhibited a high ACL-RSI score at the end of rehabilitation. Although this outcome may be influenced by multiple factors—including favorable physical recovery, young age, competitive personality traits, and a supportive environment—the cognitive engagement and attentional distraction mechanisms inherent in DL may have contributed, at least in part, to enhanced psychological readiness [65]. In this case study, the athlete exhibited a high ACL-RSI score at the end of rehabilitation, suggesting that DL played a role in improving psychological readiness. In a similar vein, studies have highlighted the benefits of DL in coping with choking under pressure during basketball free throws [66].

The cognitive demands of DL training may also serve as a distraction from pain or discomfort, shifting the athlete‘s focus toward task execution rather than injury. In addition, by switching all the time and not giving too much emphasis on previous trials, the judging of their own performances is reduced and may bring the athletes’ brains into states that are similar to meditative ones [12,67]. It seems that DL leads to meditative-like states that are described with “being in the moment” rather than planning what to do or remembering what not to do. In traditional ACL injury prevention programs, clinicians often use explicit, repetitive instructions to reinforce correct hip, knee, and ankle positioning, with the goal of improving knee joint control during standing, cutting, jumping, and landing [68].

However, such direct instructional strategies may inadvertently heighten an athlete’s fear of failure and pain, leading to negative self-comparisons [69,70]. Additionally, excessive focus on one’s own movement mechanics may hinder motor learning and performance, interfering with skill acquisition [68]. In contrast, DL uses variable instructions or encourages individuals to find new solutions by themselves to communicate goal-related information more effectively, making it a preferred approach for teaching and optimizing motor performance across different skill levels [71].

DL interventions not only enhance cognitive and motor engagement but also improve task performance and satisfaction [72]. This suggests that variability and adaptability in practice are more critical than rigid, repetitive drills, particularly in the context of injury prevention and rehabilitation. In this case study, qualitative feedback from the athlete revealed increased confidence and a reduced focus on the injured knee during training. These findings align with prior research suggesting that DL fosters psychological resilience, further supporting its role in enhancing mental preparedness and reducing fear of re-injury [10].

Notably, while a recent randomized controlled trial has validated the short-term (8-week) positive effects of DL on functional, biomechanical, and psychological outcomes in athletes following ACL reconstruction, the present study extends this preliminary evidence through long-term, ecologically grounded follow-up [17].

This study employed a single-case design, which, while traditionally seen as limited in generalizability, aligns with the principles of nonlinear pedagogy and differential learning. It offers ecologically valid insights into individualized, adaptive processes in post-ACL rehabilitation. Rather than seeking statistical generalization, the aim is to highlight complex behavioral trajectories. This approach supports the development of practice-oriented knowledge through structured single cases, advocating for context-sensitive methodologies in rehabilitation and sport-specific recovery [14]. Future research may consider employing randomized controlled trials with larger sample sizes to compare DL with traditional repetitive practice, quantify the degree of movement variability introduced during training (e.g., number of variations per exercise), and incorporate electromyography and motion-capture technologies to evaluate neuromuscular adaptations.

## 4. Conclusions

This case study demonstrates that integrating DL principles throughout ACL rehabilitation may enhance neuromuscular control, psychological readiness, and functional performance, supporting a successful return to sport. DL’s emphasis on movement variability aligns with the complex demands of sport-specific rehabilitation and offers a promising complement to conventional protocols. While generalizability remains limited, the findings underscore DL’s potential to optimize rehabilitation outcomes and warrant further investigation through larger, controlled trials within diverse athletic populations.

## Figures and Tables

**Table 1:** Physical performance monitoring data.

Test	Personal Best	Test	Personal Best
1RM Half Squat (kg)	145	Diagonal single-leg rebound jump—Left (RSI)	0.95
1RM Bench Press (kg)	94	0–10 sprint time (s)	1.68
Countermovement Jump (cm)	44.7	0–25 sprint time (s)	3.59
Drop Jump@30 cm (RSI)	2.05	505 Agility Test—Right (s)	4.07
Single Leg Countermovement Jump—Right (cm)	32.4	505 Agility Test—Left (s)	4.11
Single Leg Countermovement Jump—Left (cm)	29.6	YoYo IR1 Test (m)	1480
Single Leg Horizontal Hop—Right (cm)	195	V-cut Test (s)	6.41
Single Leg Horizontal Hop—Left (cm)	187	Game Peak Acceleration (m/s)	4.25
Diagonal single leg rebound jump—Right (RSI)	0.85	Game Peak Deceleration (m/s)	−4.21

**Table 2:** Examples of movement variations based on DL principles.

Geometry	Velocity
Head back and forward	The upper body largely faster than the lower body
Simultaneous swing straight arms	The right lower body slightly faster than the left lower body
Look to the right	The left ankle slightly faster than the right
Crossed arms	The right hip largely faster than the left
Arms stretched forward	Ankles slightly faster than hips
Left scapula retracted	Hips largely faster than ankles
Scapula depressed	Eccentric phase slightly faster than the concentric phase
Trunk tilted back and forward	Concentric phase largely faster than the eccentric phase
Hands on hip	Decreasing velocity
Right hand behind back	Interchangeable velocity

**Table 3:** Rehabilitation training methods based on DL.

Phase	Objectives	Next Stage Criteria		Content
Pre-operative (25 days)	Diminish inflammation, swelling and pain			DW DM-MC-FPE
Early Rehabilitation (29 days)	Restore full passive knee extension (Immediate postoperative phase) Maintain full passive knee extension (≥0 to 5–7 hyperextension)	Immediate postoperative sub-phase Quadriceps control (ability to perform a good quadriceps set and straight leg raises) Full passive knee extension PROM: 0–90° Minimal joint effusion Independent ambulation	Early rehab sub-phase Active ROM 0–115° Minimal to no knee joint effusion No joint line or patellofemoral pain	DM-MC-FPE
Mid Rehabilitation (44 days)	Full knee ROM (0–125°)	AROM 0–125° No pain or effusion SL hop test 80% of contralateral leg		DW DM-MC-FPE
Late Rehabilitation (66 days)	Normalize lower extremity strength	Full ROM SL hop test 85% of the contralateral leg Good control of SL Squat		DR-ST-P
Return to training (109 days)	Gradual return to fully unrestricted sport	Return to Sport-Specific Skills sub-phase Full ROM Good control of SL landing from a 40 cm box Good control of 50° COD to either side SLCMJ 85% of the contralateral leg <4.13 s (0–25 m sprint time)	Return to Basketball Practice sub-phase SL hop test 90% of the contralateral leg >SL Hop Left 168 cm SLCMJ 90% of the contralateral leg >YOYO 1330 m <6.41 s (V-cut test) <3.59 s (0–25 m sprint time)	DR-ST-P DSSD RBP FBP
Return to PLAY		ACL-RSI Score of 85.2% and IKDC of 95.4% Knee Outcome Survey Activities of Daily Living Scale of 95.7% interlimb asymmetries below 10% in single hop and triple hop		

Legend: ROM = Range of Motion; COD = Change of Direction; SL = Single Leg; CMJ = Countermovement Jump; IKDC = International Knee Documentation Committee subjective knee evaluation score; PROM = Passive Range of Motion; RSI = Reactive Strength Index; YOYO = Yo-Yo Intermittent Recovery Test; DW = Differential Walking; DM-MC-FPE = Differential Mobility, Motor Control, and Functional Patterning Exercises; DR-ST-P = Differential Running, Strength Training, and Plyometrics; DSSD = Differential Sport-Specific Drills; RBP = Reduced Basketball Practice; FBP = Full Basketball Practice.

## Data Availability

The data presented in this study are available on request from the corresponding author. The data are not publicly available due to privacy.

## Abbreviations

The following abbreviations are used in this manuscript:

ACL	Anterior Cruciate Ligament
ACLR	ACL Reconstruction
DL	Differential Learning
CMJ	Countermovement Jump
IKDC	International Knee Documentation Committee
RSI	Reactive Strength Index
Yo-Yo IR1	Yo-Yo Intermittent Recovery Test Level 1
TQR	Total Quality Recovery
sRPE	session Rating of Perceived Exertion
TL	Training Load
HRV	Heart Rate Variability
RHR	Resting Heart Rate
ROM	Range of Motion
BMI	Body Mass Index
